# Unveiling the flames: macrophage pyroptosis and its crucial role in liver diseases

**DOI:** 10.3389/fimmu.2024.1338125

**Published:** 2024-02-06

**Authors:** Linghong Ni, Dandan Chen, Yanping Zhao, Rongxia Ye, Peng Fang

**Affiliations:** Department of Infectious Diseases, The First Affiliated Hospital of Zhejiang Chinese Medical University (Zhejiang Provincial Hospital of Chinese Medicine), Hangzhou, Zhejiang, China

**Keywords:** macrophage, pyroptosis, liver disease, innate immunity, therapy

## Abstract

Macrophages play a critical role in innate immunity, with approximately 90% of the total macrophage population in the human body residing in the liver. This population encompasses both resident and infiltrating macrophages. Recent studies highlight the pivotal role of liver macrophages in various aspects such as liver inflammation, regeneration, and immune regulation. A novel pro-inflammatory programmed cell death, pyroptosis, initially identified in macrophages, has garnered substantial attention since its discovery. Studies investigating pyroptosis and inflammation progression have particularly centered around macrophages. In liver diseases, pyroptosis plays an important role in driving the inflammatory response, facilitating the fibrotic process, and promoting tumor progression. Notably, the role of macrophage pyroptosis cannot be understated. This review primarily focuses on the role of macrophage pyroptosis in liver diseases. Additionally, it underscores the therapeutic potential inherent in targeting macrophage pyroptosis.

## 1 Introduction

The liver plays a crucial role in facilitating essential physiological functions within the human body. It serves as both a metabolic organ, regulating numerous nutrients and chemical substances, and a substantial immune organ, housing a significant population of innate immune cells (1). Over recent decades, significant progress has been made in combating liver diseases. Notably, the treatment landscape of viral hepatitis, such as hepatitis C, has witnessed transformative progress with the advent of direct-acting antivirals, leading to the successful eradication of the disease (2). Similarly, nucleotide/nucleoside analogs have demonstrated efficacy in managing hepatitis B (3). However, liver disease remains among the top ten leading causes of global mortality (4). Thus, delving into the pathogenesis of liver diseases and developing targeted therapeutic interventions holds immense significance. Such endeavors not only foster the evolution of therapeutic modalities but also hold the potential to alleviate the socio-economic burden posed by liver diseases.

## 2 Macrophages in liver

Macrophages are a vital component of the immune system, serving as the body’s primary defense against pathogens and foreign invaders in the innate immune system (5). These versatile cells are part of the mononuclear phagocyte system and are found in various tissues throughout the body (6). The liver, an essential organ within the immune system, plays a pivotal role by filtering and processing blood, making it a central hub for immune-related activities. Furthermore, the liver serves as a key site for immune cell interactions, the initiation of immune responses, and the establishment of immune tolerance (7). Moreover, the liver is the organ with the highest number of macrophages among solid organs, accounting for approximately 90% of the total macrophage count in the body. In the liver of healthy rodents, this ratio is approximately 20 to 40 macrophages for every 100 liver cells (8). Including Kupffer cells (KCs), liver macrophages patrol within the liver, acting as the first line of defense in detecting and clearing pathogens from the blood and intestines. These macrophages possess a range of scavenger receptors, toll-like receptors, complement receptors, etc., enabling them to detect, bind, and internalize pathogens. Simultaneously, they produce cytokines and chemokines (9). Macrophages influence the proliferation and function of T cells by strongly expressing Programmed Cell Death 1 (PD-1) and Programmed Death-Ligand 1 (PD-L1), and by engaging in direct interactions with T cells.(Liver inflammation abrogates immunological tolerance induced by KCs). Additionally, the interaction between liver macrophages and Tregs is crucial for the production of IL-10 and subsequent induction of immune tolerance by systemic T cells to liver-derived antigens (10).

### 2.1 Macrophage population in the liver

**Figure 1:**
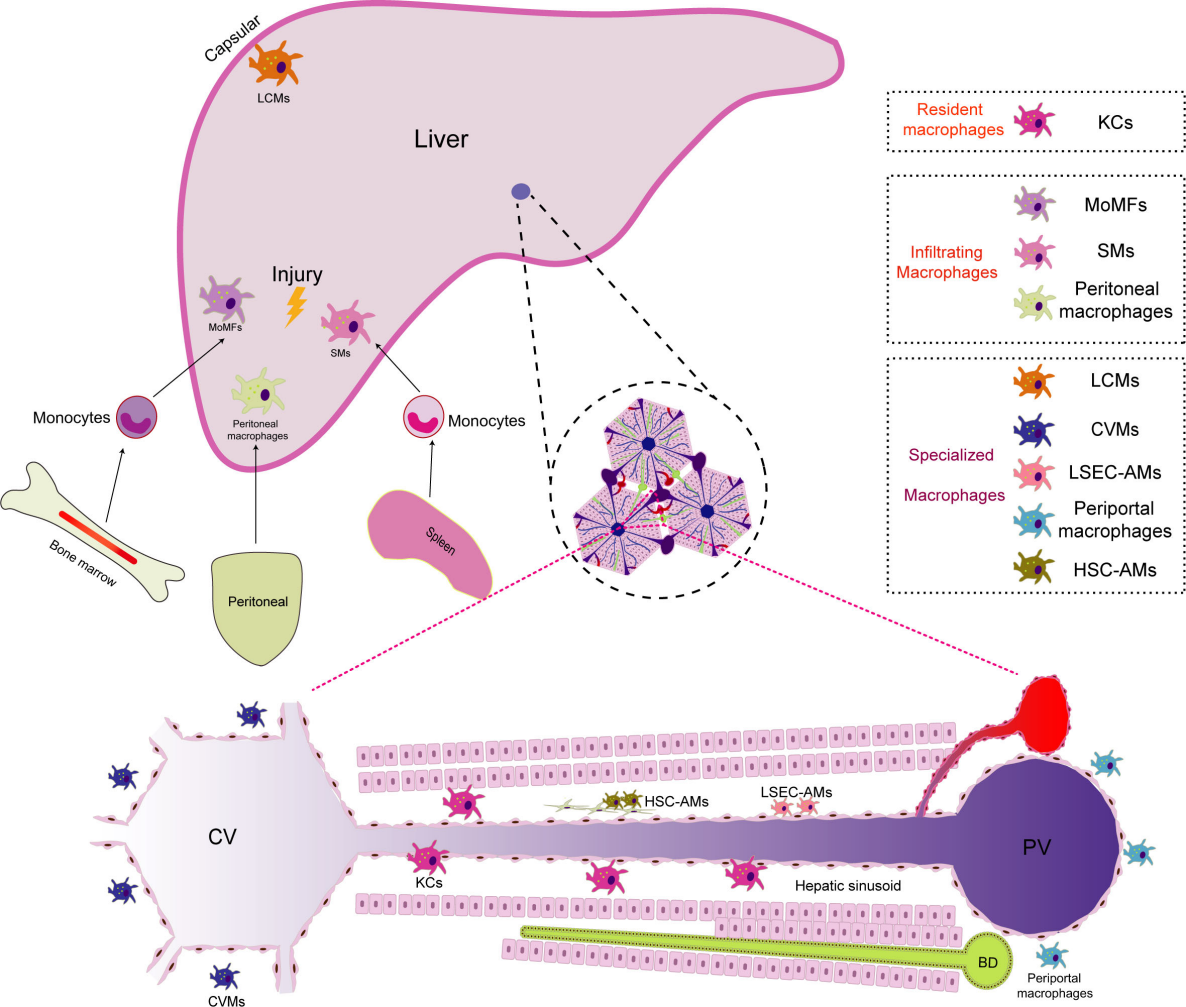
The distribution of macrophage populations in the liver. Macrophages in the liver are categorized into different subgroups due to their diverse developmental origins, specific locations, and unique microenvironments. Within the liver, there are primarily three macrophage subgroups (1):.Resident macrophages: Kupffer cells (KCs) serve as the predominant resident macrophages in the liver. They are primarily situated within the hepatic sinusoids, where they play a crucial role in regulating immune responses and safeguarding the body against infections (2). Infiltrating macrophages: In instances of liver injury, infiltrating macrophages supplement the resident macrophages. Monocyte-derived macrophages (MoMFs) originate from monocytes released from the bone marrow into the bloodstream, subsequently differentiating upon entering the liver. Peritoneal macrophages have the unique ability to traverse mesothelial cells, thus facilitating liver regeneration. Additionally, splenic macrophages (SMs) are transported to the liver, contributing to immunomodulatory functions during liver injuries (3). Specialized macrophages: Owing to their distinct locations within the liver, specialized macrophages differentiate into unique subpopulations. Periportal macrophages are positioned near portal triads, where they monitor the blood supply from the gastrointestinal tract and filter potential threats. Central vein macrophages (CVMs) are found near central veins, where they play a role in clearing toxins. Liver capsule macrophages (LCMs) reside within the liver capsule and are responsible for surveilling nearby threats. Liver sinusoidal endothelial cell (LSEC)-associated macrophages (LSEC-AMs) directly interact with liver sinusoidal endothelial cells, influencing blood flow and immune responses. Hepatic stellate cell (HSC)-associated macrophages (HSC-AMs) are situated in close proximity to HSCs, impacting their activation and involvement in the fibrosis process.

Due to diverse developmental origins, specific localizations within the liver, and distinct microenvironments, macrophages within the liver are classified into various subgroups. Each subgroup of macrophages exhibits unique characteristics and functions, collectively known as the heterogeneity of liver macrophages (11, 12). This heterogeneity mirrors the intricate and dynamic immune environment, allowing liver macrophages to adapt and efficiently respond to a wide range of physiological and pathological conditions within the organ16. According to current research, the normal liver comprises at least three cellular groups composed of the mononuclear-macrophage lineage (1): Resident macrophages: KCs (2); Infiltrating macrophages: including monocyte-derived macrophages(MoMFs), peritoneal macrophages, and splenic macrophages(SMs) (3); Specialized macrophages: such as central vein macrophages(CVMs), pericentral macrophages, liver capsule macrophages(LCMs), liver sinusoidal endothelial cell (LSEC)-associated macrophages(LSEC-AMs), hepatic stellate cell(HSC)-associated macrophages(HSC-AMs) (12–14). The liver macrophage population is highly dynamic. When exposed to external stimuli such as injury, infection, or inflammation, liver macrophages exhibit versatile responses. They get activated by growth factors, cytokines, or pathogen-associated molecular patterns (PAMPs), triggering proliferative growth and consequently increasing the number of macrophages in specific regions of the liver (15, 16). Guided by the encountered stimuli, proliferating macrophages undergo differentiation, giving rise to subtypes with distinct functions, notably the pro-inflammatory (M1-like) or anti-inflammatory (M2-like) phenotypes (17). Moreover, when the liver is challenged by infection, injury, or inflammation, signaling molecules like cytokines and chemokines orchestrate the journey of monocytes from the bone marrow into the bloodstream (18). These signals then guide monocytes towards the liver. Upon reaching the liver’s vascular system, these monocytes are captivated by specific adhesion molecules expressed on the vascular endothelial cells within the liver. The adhesion molecules, including selectins and integrins, play a pivotal role in the initial adhesion of monocytes to endothelial cells. In the intricate interplay between these adhesion molecules and chemokines, monocytes are steered to traverse the endothelial barrier and enter the liver tissue, finding their niche in specific liver regions. Upon entry into the liver, monocytes undergo differentiation into mature macrophages, assuming specific functions dictated by their location and the unique hepatic microenvironment (19). Notably, during liver injury, peritoneal macrophages demonstrate the remarkable ability to directly traverse the interstitial cells. For example, in mice with sterile focal liver injury, a specific subgroup of peritoneal macrophages (GATA6^+^) infiltrates liver tissue by penetrating the liver capsule through the peritoneal cavity and responds to liver damage. In mice with carbon tetrachloride (CCl4)-induced hepatotoxic liver injury, GATA6^+^ peritoneal macrophages similarly infiltrate into liver tissue. They promote liver tissue repair by phagocytosing necrotic cells and activating into M2-type macrophages (20). The spleen, serving as a reservoir and distributor of monocytes, also contributes replenishing monocytes to the macrophage population in the liver. For example, in mice with CCl4-induced liver fibrosis, a specific subgroup of CD11b^+^CD43^hi^Ly6C^lo^ monocytes expands in the spleen. These monocytes are actively recruited to the liver, undergo a phenotypic shift toward macrophages, and promote the accumulation of Ly6C^lo^MoMFs. Ultimately, it exacerbates liver fibrogenesis by activating HSCs. This phenomenon may partially explain the pathophysiological connection of the liver-spleen axis (21). The dynamic evolution of the macrophage population in the liver is profoundly influenced by the hepatic microenvironment, immune responses, and pathological conditions. This adaptive dynamism is pivotal for the liver macrophage population to effectively respond to an ever-changing environment, promote immune responses, and maintain hepatic homeostasis (22, 23) (**Figure 1**).

## 3 Pyroptosis

Pyroptosis represents a distinct form of programmed cell death characterized by the formation of pores within the cell membrane, the ensuing release of intracellular contents, nucleus condensation, and eventual cell lysis (24). The term “pyroptosis” originates from the fusion of “pyro” akin to fireworks, illustrating the inflammatory attributes of this process, and “ptosis” implying descent consistent with other programmed cell death pathways. The trajectory of the pyroptosis revelation has traversed a convoluted historical path. In 1992, Zychlinsky’s first observations revealed pyroptosis in macrophages infected with Shigella flexneri, a gram-negative bacterium. Initially, this phenomenon was erroneously classified as a variant of apoptosis due to shared characteristics such as caspase dependence, DNA damage, and nuclear pyknosis (25). However, subsequent investigations revealed its divergence from apoptosis. By 2001, it was established as a caspase-1-dependent pro-inflammatory programmed cell death (26). In 2005, the term “pyroptosis” was coined to distinctly characterize this pro-inflammatory programmed cell death in contrast to apoptosis, which is non-inflammatory (27).

### 3.1 Mechanisms of pyroptosis

**Figure 2:**
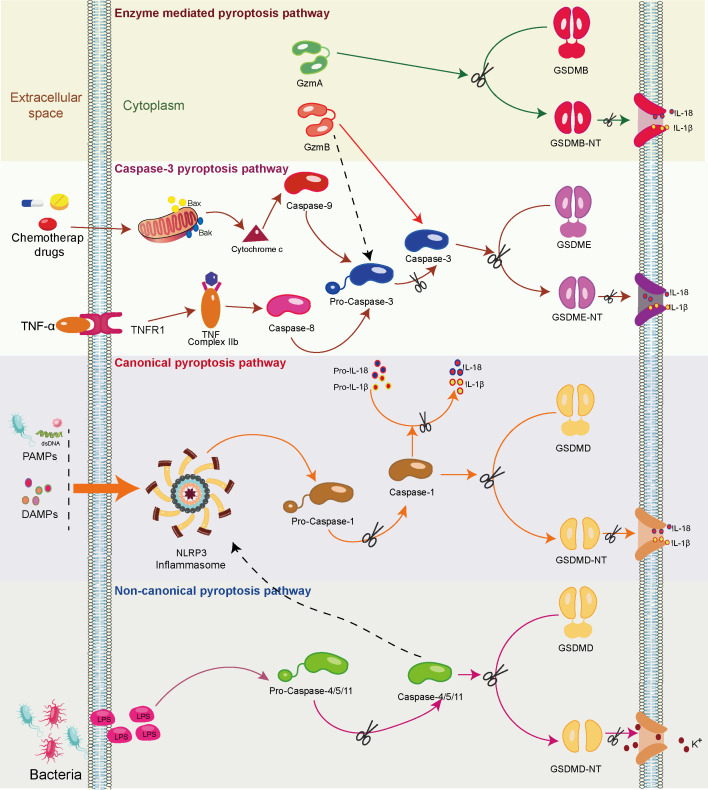
The mechanism of the pyroptosis pathway. Upon recognizing pathogen-associated molecular patterns (PAMPs) and damage-associated molecular patterns (DAMPs), the canonical pyroptosis cascade unfolds. Initiation prompts the assembly of the inflammasome complex, culminating in the activation of the NOD-like receptor protein 3 (NLRP3) inflammasome (Caspase-1). Activated Caspase-1 cleaves gasdermin D (GSDMD), yielding a peptide containing the N-terminal active domain and inducing cell membrane perforation. Additionally, activated Caspase-1 processes precursor forms of IL-1β and IL-18 into active IL-1β and IL-18, which are subsequently released through membrane pores. In the non-canonical pyroptosis pathway, lipopolysaccharides (LPS) activate caspase-11/4/5 proteins, leading to GSDMD cleavage and ensuing K^+^ efflux. Moreover, caspase-11/4/5 indirectly facilitates IL-1β/IL-18 maturation and secretion through the NLRP3/caspase-1 pathway in some cells. In the caspase-8/caspase-3-mediated pyroptosis pathway, the activation of intracellular TNF complex IIb by TNF-α instigates caspase-8 activation. This, in turn, cleaves downstream caspase-3, ultimately triggering the activation of GSDME and resulting in pyroptosis. In addition, chemotherapeutic drugs prompt the release of mitochondrial cytochrome C, subsequently activating caspase-9 and its downstream caspase-3, ultimately leading to pyroptosis. In enzyme-mediated pyroptosis, granzyme A (GzmA) directly cleaves Gasdermin B (GSDMB), triggering pyroptosis. Meanwhile, GzmB triggers caspase-3/Gasdermin E (GSDME)-mediated pyroptosis. In addition, GzmB directly cleaves GSDME, further driving pyroptosis.

Further contributing to pyroptosis initiation is the activation of inflammasomes, wherein the NOD-like receptor protein 3 (NLRP-3) inflammasome, when engaged in an inflammatory milieu, activates gasdermin D (GSDMD) in a caspase-1-, caspase-4/5-, or caspase-11-dependent manner (28). Subsequently, GSDMD undergoes cleavage, yielding a peptide containing an N-terminal domain (GSDMD-NT), inducing the formation of pores (1.1 to 2.4 nm in diameter) across the cell membranes, leading to cell swelling and rupture. Notably, this event leads to the extracellular release of numerous pro-inflammatory factors, such as IL-1β and IL-18. This, in turn, triggers pro-inflammatory signals, recruits inflammatory cells, and amplifies the overall inflammatory response (29) (**Figure 2**).

### 3.2 Two other novel mechanisms of pyroptosis

#### 3.2.1 Caspase-3-mediated pyroptosis pathway

Caspase-3 has conventionally been recognized as an executor of apoptosis. However, recent studies indicate its involvement in inducing pyroptosis as well (30). In cells expressing high levels of gasdermin E (GSDME), tumor necrosis factor (TNF)- or chemotherapy-induced caspase-3-mediated apoptosis can be redirected toward pyroptosis. This intriguing shift involves the targeted cleavage of GSDME by caspase-3, generating a transmembrane GSDME-N fragment that effectively elicits pyroptosis (30). During chemotherapy-induced pyroptosis, the activation of mitochondrial BAK/BAX prompts the release of cytochrome C. Subsequently, caspase-9 and, consequently, caspase-3 are activated, leading to pyroptosis (31). Notably, in macrophages, inhibiting caspase-3 and silencing GSDME have been shown to significantly block TNF-induced pyroptosis (32). In TNF-induced pyroptosis, TNF-α-activated intracellular TNF complex IIb activates caspase-8. This, in turn, triggers downstream activation of caspase-3, ultimately leading to pyroptosis in cells with high GSDME expression (33) (**Figure 2**).

#### 3.2.2 Enzyme-mediated pyroptosis pathway

Cytotoxic lymphocyte-mediated immune processes crucially rely on granzymes. Notably, a pivotal discovery in 2020 unveiled the rapid activation of caspase-3 in target cells by CAR-T cells, facilitated through the release of granzyme B (GzmB) from cytotoxic lymphocytes. This activation subsequently initiates the caspase-3/GSDME-mediated pyroptosis pathway, leading to extensive pyroptotic events (34). Recently, it has been found that GzmB can directly cleave GSDME in tumor cells, thereby inducing pyroptosis and further activating anti-tumor immune responses (35). Furthermore, cytotoxic lymphocyte-derived granzyme A (GzmA) has been found to cleave GSDMB, triggering pyroptosis and fostering antitumor immune responses (36, 37) (**Figure 2**).

## 4 The impact of macrophage pyroptosis on liver immunity

**Figure 3:**
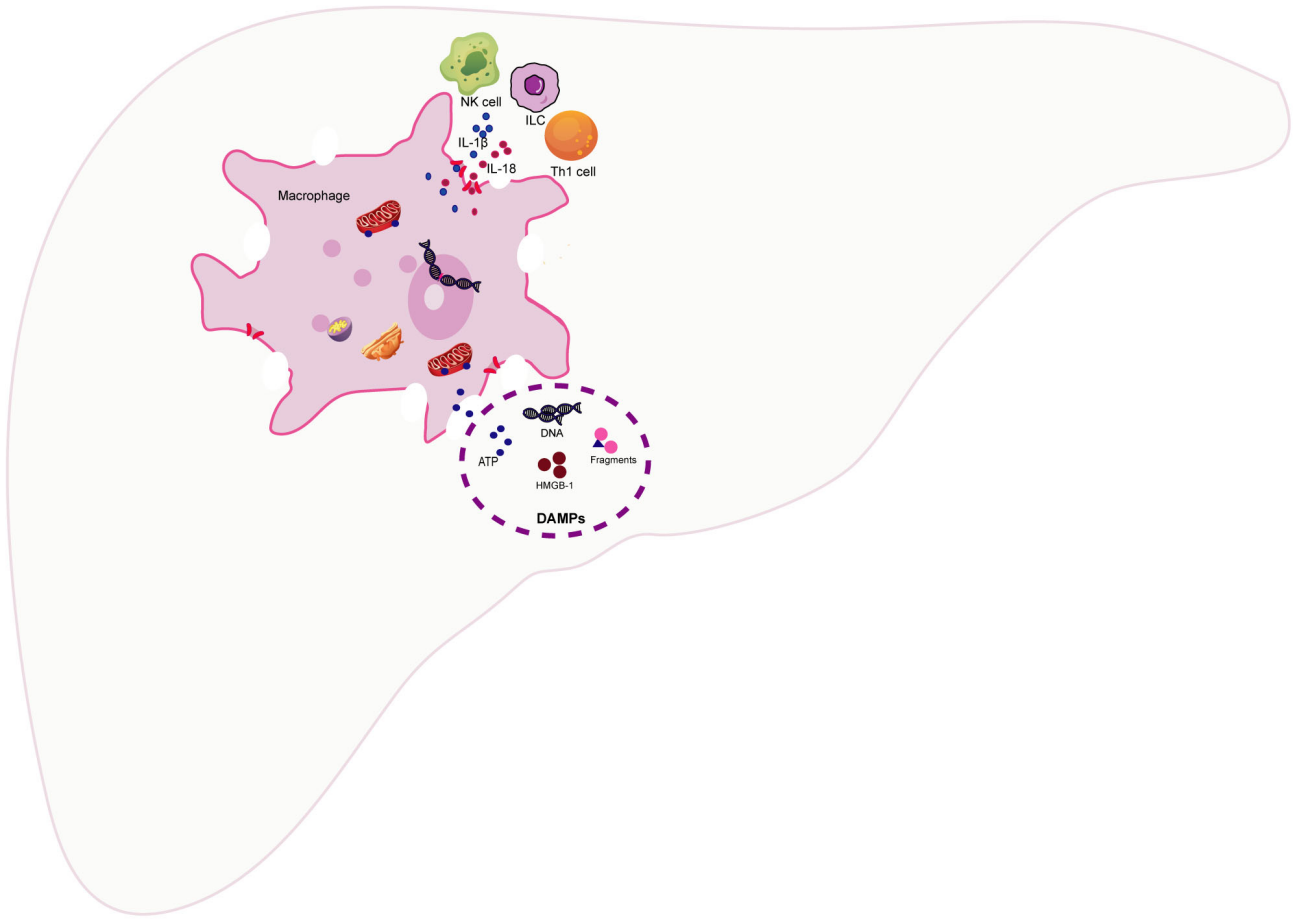
The impact of macrophage pyroptosis on liver immunity. The pyroptosis of macrophages has a predominant impact on liver immunity through the following two ways (1).The pyroptosis of macrophages leads to the active release of a substantial amount of pro-inflammatory cytokines, such as Interleukin-1β(IL-1β) and interleukin-18(IL-18), to participate in the recruitment and differentiation of T helper 1 (Th1) cells, as well as the activation of natural killer (NK) cells and innate lymphoid cells (ILC) (2)..Macrophage pyroptosis results in cell membrane swelling, rupture, and the release of cellular contents, such as nuclear and mitochondrial DNA, high mobility group box-1 (HMGB-1), adenosine triphosphate (ATP), and fragments of cellular organelles. Once released into the extracellular space, these self-structures from separated cells can be recognized as damage-associated molecular patterns (DAMPs). DAMPs initiate pro-inflammatory responses by interacting with cell receptors, including pattern recognition receptors (PRRs). Furthermore, they elicit immune responses in myeloid cells and dendritic cells, thereby regulating the activation of innate immune cells and programmed cell death.

Macrophages serve as monitors of the liver immune response. Therefore, when macrophages undergo pyroptosis, it undoubtedly disrupts liver immune homeostasis and triggers a strong immune response. Macrophage pyroptosis may impact liver immunity primarily through two mechanisms: 1). Pyroptotic macrophages actively release a substantial number of proinflammatory cytokines, including IL-1β and IL-18. Among these, the pleiotropic proinflammatory cytokine IL-1β can stimulate the expression of proinflammatory cytokines and recruit neutrophils to liver tissue, thereby amplifying the inflammatory response. Simultaneously, IL-1β can mimic T lymphocytes, induce Th17 differentiation, and recruit inflammatory cells (38). KCs are the primary producers of IL-1β and IL-18. Moreover, KCs enhance the production of IFN-γ and TNF-α by Th1 and natural killer(NK) cells in a TLR9-dependent induction and NALP3-ASC-caspase-1-dependent manner (39). IL-18 and IL-1 enhance the recruitment and differentiation of T helper 1 (Th1) cells, as well as the activation of NK cells and innate lymphoid cells (ILC), by inducing the production of IFNγ. The overexpression of IL-18 induces FasL expression on NK cells and CD4^+^ T cells, while also upregulating Fas expression on hepatocytes, thereby sensitizing them to NK cytotoxicity (40). In mice with autoimmune hepatitis (AIH), the inhibition of NLRP3 inflammasome-mediated pyroptosis-derived IL-1β led to a decreased accumulation of CD68-positive cells and CD11b-positive cells in liver tissue (41). In acetaminophen (APAP)-induced acute liver failure (ALF), IL-1β and IL-18 produced by KCs increased IFN-γ and TNF-α production by Th1 and NK cells in a TLR9/NALP3/ASC/Caspase-1-dependent manner. IL-18-deficient mice exhibited resistance to APAP-induced ALF, and blocking IL-1β with neutralizing antibodies reduced the severity of ALF (42). 2). In addition, macrophage pyroptosis causes the cell membrane to swell and rupture, releasing cell contents, such as nuclear and mitochondrial DNA, nuclear proteins (e.g., high mobility group box-1, HMGB-1), purine nucleotides (e.g., adenosine triphosphate, ATP), fragments of organelles, etc. (43). Once released into the extracellular environment, these self-isolated structures resulting from cell separation are recognized as damage-associated molecular patterns (DAMPs) (44). DAMPs are typically present intracellularly, playing various roles in maintaining internal balance. Once translocated to the extracellular space, they are recognized by other cells through interactions with pattern recognition receptors (PRRs) and other cell receptors. Subsequently, stress response mechanisms are upregulated, converging to form a positive feedback loop that leads to tissue damage and inflammation (45). In mice with cyclophosphamide (CPA)-induced liver injury, the release of DAMPs, such as HMGB1, heat shock protein 60 (HSP60), and glucose-regulated protein 94 (Grp94), increases. This results in the activation of the TLR4-NFκB signaling pathway, thereby exacerbating the inflammatory response in the liver (46). Taking HMGB1 as an example, its release from the canonical pyroptosis pathway can aggravate acute liver injury(ALI) induced by APAP in mice (47). Compared to wild-type mice, inflammation and neutrophil recruitment are significantly reduced in HMGB1-deficient mice (48). Furthermore, HMGB1-induced hepatocyte pyroptosis directly amplifies the inflammatory response, exacerbating acute-on-chronic liver failure (ACLF) in rats (49). Infiltrating myeloid and lymphoid cells in the liver also respond to the stimulation of DAMPs (50). Therefore, the activation of PRRs triggered by DAMPs not only initiates proinflammatory signals to amplify the inflammatory response but also triggers an immune response in myeloid and dendritic cells, thereby regulating the activation of innate immune cells and parenchymal cell death (51) (**Figure 3**).

## 5 The role of macrophage pyroptosis in liver diseases

The role of pyroptosis in liver diseases has garnered increasing interest. Excessive pyroptosis initiates a cascade of intense inflammatory responses, resulting in substantial damage to hepatocytes and the overall structure and function of the liver (52). Numerous studies on the role of macrophage pyroptosis in liver disease have been reported by researchers, which have been summarized in **Table 1**. Notably, macrophage pyroptosis plays a crucial role in various liver diseases (58).

**TABLE 1:** The role and mechanism of macrophage pyroptosis in liver disease.

Animal	Disease	Model	Macrophage pyroptosis	Mechanisms	Year and citation
Mouse	Sepsis	CLP	+	NA	2020 (53)
Mouse	Acute Liver Injury	LPS	+	P2X7R/NLRP3 pathway↑; GSDMD-N↑	2022 (54)
Mouse	Acute Liver Injury	LPS/D-GalN	+	MaR1↓; NLRP3↑; GSDMD-N↑; IL-1β↑	2022 (55)
Mouse	Acute Liver Injury	Triptolide	+	Caspase3↑; GSDME↑ IL-1β↑	2023 (56)
Mouse	Liver ischaemia -reperfusion injury	(70%) warm liver IR	+	Caspase-11↑; NF-KB↑ NLPR3↑	2021 (57)
Mouse	Liver ischaemia-reperfusion injury	(70%) warm liver IR	+	ApoA-1↓; Caspase-1↑GSDMD↑; TLR4-NF-κB pathway↑	2023 (58)
Human/Mouse	Liver ischaemia -reperfusion injury	Fatty liver samples (reperfusion after resection) /Fatty liver IR model	+	Caspase 6/NR4A1/SOX9 pathway↑ NLRP3↑; Caspase-1↑	2023 (59)
Mouse	Liver ischaemia-reperfusion injury	(70%) warm liver IR	+	TLR4 / MyD88 / NF-κB pathway↑;HMGB1↑; Caspase-1↑	2023 (60)
Mouse	Liver ischaemia-reperfusion injury	warm liver IRI	+	HMGB1↑; Caspase-1↑; GSDMD↑	2019 (61)
Mouse	Liver ischaemia-reperfusion injury	warm liver IRI	+	STING↑; Ca2+↑; Caspase-1↑; GSDMD↑	2022 (62)
Human/ Mouse	Liver ischaemia-reperfusion injury	liver IRI samples 75% warm ischemia	+	Ikaros↑; AMPK↑; SIRT1↓; Caspase-1↑; GSDMD↑	2022 (63)
Mouse	Alcoholic Liver Disease	Ethanol+EHFD	**+**	NF-κB–NLRP3 pathway↑; Caspase-1↑; GSDMD↑	2021 (64)
Mouse	Alcoholic Liver Disease	Ethanol	**+**	METTL3↑; NLRP3↑; Caspase-1↑; GSDMD↑	2023 (65)
Mouse	Non-alcoholic fatty	CDAA diet	NA	NLRP3↑; IL-1β↑;	2014 (66)
Mouse	Non-alcoholic steatohepatitis/ Non-alcoholic fatty liver disease	MCD/HFD diet	NA	Srebf1↑; NF-κB ↑; GSDMD↑;	2018 (67)
Mouse	Non-alcoholic fatty liver disease	HFD diet	+	Caspase-11↑; NLRP3↑; GSDMD↑;	2023 (68)
Mouse	Liver Fibrosis	CCl^4^/BDL	+	lnc-Lfar1↑; NF-ĸB↑;NLPR3↑; Caspase-1↑; GSDMD↑	2020 (69)
Mouse	Liver Fibrosis	CCl^4^	+	METTL3/MALAT1/PTBP1/USP8/TAK1 axis↑NF-κB↑; NLRP3↑; Caspase-1↑; GSDMD↑;	2021 (65)
Mouse	Acute Liver Failure	D-GalN/LPS	+	NLPR3↑; Caspase-1↑; ASC↑; GSDMD↑	2021 (70)
Mouse	Acute on chronic liver failure	CCl4+D-GalN/LPS	NA	GAL3↓; NLPR3↑; Caspase-1↑; GSDMD↑	2022 (71)

NA , Not Available; ” ↑ ”, function or proliferation promoted ; “ ↓ ”, function or proliferation suppressed; ”+”, Macrophage pyroptosis occurred.

### 5.1 Acute liver injury

Acute liver injury (ALI) encompasses hepatocyte necrosis and acute liver inflammation caused by endotoxins, certain drugs and their metabolites, and various physical or chemical agents. This can lead to liver dysfunction and even acute liver failure (72). Lipopolysaccharides (LPS), D-galactosamine (D-GalN), thioacetamide (TAA), and APAP are commonly used compounds to establish experimental models of ALI (73, 74). ALI is intricately linked to disruptions in innate immunity and acute hepatocyte injury, with macrophage pyroptosis playing an important role in this process (75). In the mouse model of cecal ligation puncture (CLP)-induced septic ALI, substantial infiltration of macrophages occurs in the liver. The overall percentage of pyroptotic cells in the liver is 18.19%, with a pyroptosis rate of 16.29% in liver macrophages (53). In LPS-induced ALI, the P2X7R/NLRP3 pathway is activated in macrophages, leading to GSDMD-N activation and pyroptosis (54). In LPS/D-GalN induced ALI model, the inhibition of macrophage pyroptosis by Maresin 1(MaR1) leads to significant reductions in NLRP3 inflammasome expression, consequently suppressing GSDMD-N and IL-1β expression, effectively tempering macrophage pyroptosis, thereby alleviating the extent of liver injury in ALI (55). Triptolide (TRI)-induced ALI triggers pyroptosis in KCs, promoting the maturation and release of inflammatory cytokines. The whole-body knockout of Caspase-3 in mice significantly mitigates liver injury in mice subjected to TRI, and GSDME knockdown prevents TRI-induced pyroptosis in KCs. Therefore, the mechanistic basis for TRI-induced pyroptosis in KCs hinges on the Caspase-3-GSDME pathway. Suppression of pyroptosis in KCs significantly attenuates TRI-induced ALI (56). In summary, macrophage pyroptosis occupies a pivotal role in diverse contexts of sepsis and drug-induced ALI. Therefore, targeting macrophage pyroptosis stands out as a promising avenue for mitigating ALI.

### 5.2 Liver ischemia/reperfusion injury

Liver ischaemia/reperfusion injury (IRI) constitutes a significant challenge in liver surgery, notably in cases involving liver transplantation, partial hepatectomy, and hypovolemic shock. The mounting prevalence of liver transplantation highlights the urgency to address IRI, which hampers postoperative liver function recovery (76). Cellular damage during IRI results directly from blood flow interruption and is further exacerbated upon organ reperfusion. This process leads to impaired ATP production, excessive reactive oxygen species (ROS) accumulation, and systemic inflammatory activation, leading to parenchymal damage and organ dysfunction (77). Despite being a significant clinical concern, the mechanism underlying liver IRI remains poorly understood, presenting a knowledge gap that requires attention. Recent insights underscore the pivotal role of macrophages in liver IRI. Simultaneously, emerging evidence indicates that pyroptosis in hepatocytes exacerbates liver damage in IRI mice (78). Considering the profound impact of pyroptosis on liver innate immune cells and the central role of macrophages in liver IRI injury (57), the role of macrophage pyroptosis in liver IRI deserves further investigation. In a mouse model of warm liver IRI, inhibition of liver macrophages by gadolinium chloride (GdCl3) significantly downregulates the expressions of caspase-11, IL-1β, and IL-18. This implies the involvement of non-canonical pyroptosis in the macrophage-driven hepatic IRI. Isoflurane intervention curbs this pyroptosis by inhibiting intracellular Ca^2+^ levels, NF-κB translocation, NLPR3 inflammasome activation, and the caspase-11-mediated non-canonical pyroptosis pathway (58). The downregulation of ApoA-1 during IRI after liver transplantation increases pyroptosis (but not apoptosis) in liver macrophages. The use of TLR4 inhibitors ameliorates this effect, suggesting a connection between the ApoA-1 pathway and the TLR4-NF-κB pathway (79). Liver steatosis accentuates IRI sensitivity. Patients with NASH experience heightened serum ALT levels after IRI, accompanied by intensified histopathological damage, pro-inflammatory mediator release, and macrophage activation. In the liver macrophages of patients with NASH, caspase-6 is significantly increased, promoting the upregulation of SOX9 and NR4A1 in the nucleus. This prompts the interaction between NR4A1 and SOX9, ultimately triggering the transcription of the downstream target gene S100A9. Consequently, the NEK7/NLRP3 inflammasome is activated and macrophage pyroptosis is induced (59). The MyD88 inhibitor TJ-5 can significantly alleviate IRI, primarily by inhibiting pyroptosis (particularly KC pyroptosis) and liver macrophage depletion. *In vitro*, TJ-5 inhibits the TLR4/MyD88/NF-κB signaling pathway. It alleviates canonical and non-canonical pyroptosis in BMDM by inhibiting the release of HMGB1 and entosis of the LPS-HMGB1 complex, respectively (60). In liver IRI, GSDMD mediates KCs pyroptosis. Inhibiting HMGB1 with glycyrrhizin attenuated both intra- and extracellular inhibition of macrophage pyroptosis, thereby alleviating liver IRI. Inhibiting HMGB1 with glycyrrhizin alleviated its mediation of macrophage pyroptosis both *in vitro* and *in vivo*, thereby reducing liver IRI (61). The STING signaling pathway regulates macrophage pro-inflammatory activation and liver IRI (62). In the IRI model, the expression of STING increases but significantly decreases after depleting liver macrophages with clodronate liposomes. *In vitro*, hypoxia-reoxygenation (H/R)-induced STING increases intracellular calcium, promoting Caspase 1-GSDMD activation in liver macrophages. This underscores how STING regulates calcium influx to mediate H/R-induced pyroptosis in liver macrophages (80). The mechanism of liver IRI in transplant recipients is associated with an elevation in liver Ikaros levels post-reperfusion, accompanied by enhanced inflammasome signaling and SIRT1 inhibition. Research on liver IRI in mice revealed that Ikaros is mainly expressed by liver-infiltrating CD11b^+^ macrophages. Verified through the CD11b^-^ DTR mouse system, Ikaros-silenced bone marrow-derived macrophages improved liver IRI and cell pyroptosis. The specific mechanism involved the Ikaros-SIRT1 axis mediated through the AMPK signaling pathway (63).

### 5.3 Alcoholic liver disease

Macrophages play a pivotal role in the progression of alcoholic liver disease (ALD), promoting steatosis, inflammation, and fibrosis. Macrophage infiltration is significantly increased in the liver of patients with ALD (81). ALD is attributed to hepatocyte death and steatosis induced by alcohol and its metabolites. In addition, direct effects such as macrophage infiltration, cytokine/chemokine transduction, and proinflammatory cytokine release are implicated in the pathological mechanism (82). Investigations into KCs isolated from the liver of ALD mice have highlighted alcohol-induced pyroptosis in these cells. Silencing the m6A enzyme methyltransferase-like 3 (METTL3) alleviates inflammatory factor release by repressing KC pyroptosis in ALD mice. This underscores the pivotal role of KC pyroptosis in ALD mice (83). Suppression of macrophages contributes to alleviating ALD-associated pathological damage. Studies have shown that cannabidiol (CBD) prevents Lieber-DeCarli ethanol diet-induced fatty liver in mice by inhibiting liver macrophage recruitment and NF-κB-NLRP3 pathway-mediated pyroptosis (64). However, it is noteworthy that macrophage depletion with clodronate liposomes neither induces liver histopathological changes nor aggravates ethanol-induced liver injury (84). Conversely, alcohol induces pyroptosis in hepatocytes through TXNIP-mediated activation of the NLRP3 inflammasome (85). To date, most studies underscore the significant correlation between ALD pathology and increased macrophage infiltration, which is accompanied by pro-inflammatory factor release. As the most widely used method for macrophage depletion, clodronate liposomes offer economic advantages and simple preparation. However, their efficacy in macrophage depletion varies greatly (usually 50%–95%), and intravenous administration may not specifically deplete liver macrophages (86). Therefore, the reliability of the findings warrants further investigation. In conclusion, the specific role of macrophage pyroptosis in ALD necessitates deeper exploration through targeted regulation of macrophage pyroptosis.

### 5.4 Non-alcoholic fatty liver disease

Non-alcoholic fatty liver disease (NAFLD) affects approximately 25% of the global population and stands as the leading cause of cirrhosis and hepatocellular carcinoma (87). NAFLD encompasses a spectrum from simple steatosis to non-alcoholic steatohepatitis (NASH). In contrast to ALD, NAFLD’s hallmark is the hepatocyte accumulation of fatty acids exceeding the liver’s lipid metabolism capacity, fostering hepatic inflammation featuring ceramide, diacylglycerol, lipid peroxides, and oxidized phospholipids (88). Liver biopsy samples from patients during bariatric surgery revealed that, compared to non-NASH patients, patients with NASH exhibit significantly higher expression of NLRP3 inflammasome-related proteins, including NLRP3, pre-IL-1β, and pre-IL-18 (66). Liver macrophages, especially KCs and monocyte-derived macrophages (MDMs), are implicated in the initiation and progression of inflammation in patients with NAFLD and NASH as well as in their corresponding animal models (89). Resident KC activation and macrophage infiltration are prominent features of NASH pathogenesis (90). Depletion of KCs hinders the transition from non-alcoholic fatty liver (NAFL) to NASH and prevents the recruitment of MDMs, significantly reducing NASH-associated inflammation (91). High-fat diet (HFD)-fed caspase-1^-/-^ mice showed improved liver steatosis compared to wild-type mice, though ALT levels did not correspondingly improve, indicating caspase-1 knockout did not alleviate hepatocyte injury (67). However, in NAFLD induced by a short-term choline-deficient amino acid (CDAA) diet, NLRP3^-/-^ mice exhibited protection from hepatomegaly, liver injury, and activated macrophage infiltration. In particular, after 4 weeks of the CDAA diet, wild-type mice developed isolated hepatic steatosis, whereas NLRP3 knock-in mice exhibited severe liver inflammation with increased infiltration of activated macrophages and early signs of liver fibrosis (89). GSMD^-/-^mice fed the methionine-choline-deficient (MCD) diet showed attenuated steatohepatitis, significantly improved liver inflammation, and decreased serum ALT levels and liver triglyceride content (67). While the canonical pathway of caspase-1-mediated pyroptosis may not be operative, the activation of the NLRP3 inflammasome and the release of pro-inflammatory factors in NAFLD suggest the potential involvement of pyroptosis in NAFLD. A follow-up study revealed that HFD upregulates NASH-related liver macrophage markers, caspase-11, and GSDMD. This prompts the cleavage and membrane expression of GSDMD-NT, signifying HFD’s role in promoting macrophage pyroptosis. Moreover, the pyroptosis of BMDMs appears more pivotal than that of liver-resident macrophages in NAFLD. HFD-induced NAFLD is primarily associated with caspase-11-GSDMD-mediated non-canonical pyroptosis. Moreover, caspase-11 promotes glycolysis and oxidative phosphorylation, thereby promoting macrophage pyroptosis (68). In summary, targeting non-classical pathways of macrophage pyroptosis holds promise as an approach to inhibiting NAFLD progression.

### 5.5 Liver fibrosis

Liver fibrosis is a prevailing outcome of the healing response to chronic liver injury, characterized by increased production and accumulation of extracellular matrix (ECM). Unchecked liver fibrosis can eventually progress to cirrhosis, organ failure, and even death99. The pivotal initiation factor in liver fibrosis is the activation and differentiation of HSCs into fibroblasts. Notably, activated HSCs predominantly contribute to synthesizing and depositing ECM proteins in response to cumulative levels of inflammatory signals100. In recent years, several studies have revealed the notable role of pyroptosis in liver fibrosis. NLRP3 and GSDMD knockout mice manifest milder liver fibrosis phenotypes (92–94). The interplay among immune cells, such as KCs, MDMs, natural killer cells, B cells, and T cells, initiates liver fibrosis. Notably, liver macrophages significantly influence the progression of fibrosis. M2-polarized macrophages directly promote the progression of liver fibrosis, while M1-polarized macrophages secrete an abundance of pro-inflammatory factors, recruit pro-inflammatory cells, activate HSCs, and perpetuate liver fibrosis (5, 89). Macrophage pyroptosis exacerbates fibrotic processes. Knockdown of the lncRNA Lfar1 in liver macrophages alleviates liver fibrosis by inhibiting LPS/ATP and LPS/Nigericin-induced macrophage pyroptosis, a result of NLRP3 inflammasome activation (69). Activation of the METTL3/MALAT1/PTBP1/USP8/TAK1 axis in liver macrophages is associated with M1-polarized macrophage pyroptosis induced by NLRP3 inflammasomes. Counteracting this axis activation curbs M1-polarized macrophage pyroptosis and reduces the degree of liver fibrosis (65). Therefore, macrophages play a critical role in the initiation and perpetuation of liver fibrosis. Targeting macrophage pyroptosis to temper pro-inflammatory factor release stands as a promising avenue for inhibiting liver fibrosis.

### 5.6 Hepatocellular carcinoma

Liver cancer, particularly hepatocellular carcinoma (HCC), is a substantial global health burden, ranking as the sixth most commonly diagnosed cancer and the fourth leading cause of cancer-associated mortality worldwide. HCC accounts for approximately 85% to 90% of all primary liver cancers (95). Despite advancements in surgery, adjuvant therapy, and liver transplantation, the survival rate for patients with HCC remains unsatisfactory, with a 5-year survival rate of approximately 20% (96). Therefore, elucidating the molecular mechanism of HCC and identifying novel therapeutic targets remains crucial. Liver macrophages intricately affect the pathogenesis of HCC. Extensive macrophage accumulation has been observed in HCC liver tissues as well as mouse models of HCC (97, 98). These macrophages are tumor-promoting cells that suppress anti-tumor immunity. During HCC progression, hepatic macrophages release pro-angiogenic factors such as transforming growth factor-β (TGF-β), vascular endothelial growth factor (VEGF), and platelet-derived growth factor (PDGF), which collectively promote tumor growth (99, 100). Pyroptosis, an inflammation-associated programmed cell death, plays a dual role in tumor progression. On the one hand, inducing pyroptosis can inhibit the proliferation and migration of tumor cells. On the other hand, excessive activation of pyroptosis leads to an inflammatory tumor microenvironment (TME), which favors tumor growth. In HCC, tumor-infiltrating macrophages can be stimulated by gasdermin-mediated pyroptosis to promote phagocytosis and anti-tumor immunity (35). Interestingly, gasdermins in HCC exhibit distinct features of immune cell infiltration. Most gasdermins exhibit positive correlations with B cells, neutrophils, and dendritic cells while displaying negative correlations with macrophages (101). These results suggest that macrophage pyroptosis mediated by gasdermins may affect anti-tumor immunity by influencing the infiltration spectrum of immune cells. Therefore, immune infiltration of macrophages is closely related to HCC. The intervention of macrophage pyroptosis could potentially alter the TME to inhibit tumor cell growth and metastasis. However, this promising research direction warrants further exploration.

### 5.7 Liver failure

Liver failure is a serious syndrome encompassing hepatic encephalopathy, severe infections, coagulopathy, and renal failure, presenting a life-threatening condition. Acute liver failure (ALF) and acute-on-chronic liver failure (ACLF) are the most common types of liver failure. ALF manifests abrupt and severe liver function impairment, whereas ACLF occurs in patients with preexisting chronic liver disease, often precipitated by an event (102, 103). Both ALF and ACLF patients present a pro-inflammatory state, characterized by local liver inflammation and systemic inflammatory response syndrome (SIRS). In this context, innate immune cells, predominantly macrophages abundant in the liver, play a key role in both host defense and inflammatory regulation. However, the rapid progression of macrophage-mediated inflammation also engenders systemic consequences, a hallmark of ALF and ACLF (104). Human umbilical cord mesenchymal stem cells (UC-MSCs) have been shown to significantly attenuate liver injury and mortality in ALF-afflicted mice. The effect is linked to UC-MSCs restraining NLRP3 and GSDMD-N expression in macrophages, thereby inhibiting pyroptosis. Nevertheless, the upstream pathway leading to pyroptosis inhibition was not investigated in this study (70). GAL3, a β-galactoside-binding lectin, has been found to be significantly increased in the serum of patients with ACLF. Mainly originating from M2-polarized liver macrophages, GAL3 promoted the beneficial liver protective effect conferred by these M2 macrophages in ACLF by inhibiting pyroptosis but not necroptosis signaling. Therefore, GAL3 can directly inhibit macrophage pyroptosis and promote the liver-protective attributes of M2-polarized macrophages by inhibiting macrophage pyroptosis, thus ameliorating liver injury (71). This provides a valuable framework for subsequent macrophage and pyroptosis intervention approaches in liver failure.

## 6 Targeted inhibition of macrophage pyroptosis in the treatment of liver diseases

Inhibiting pyroptosis, particularly in immune cells, is an appealing avenue for treating liver diseases. Targeted therapeutic approaches utilizing specific inhibitors for pyroptosis have also garnered considerable attention. VX-765, an oral interleukin-converting enzyme (ICE)/caspase-1 potent and selective competitive inhibitor, can inhibit the secretion of IL-1β in cells stimulated by LPS (105). Meanwhile, as an inhibitor of the pivotal factor caspase-1 in the canonical pyroptosis pathway, VX-765 has demonstrated therapeutic potential in the context of liver diseases (106). Pre-administration of upconversion nanoparticles (UCNs) to mice before liver IRI depletes KCs through pyroptosis, effectively mitigating liver damage during IRI events. Following VX-765 intervention, which rescued UCN-induced KCs pyroptosis, the liver’s protective response to UCN was diminished. This indicates that KCs play an exacerbating role in liver IRI. Furthermore, despite the inhibition of the canonical pathway of macrophage pyroptosis, it persists in exacerbating liver IRI. This suggests that the immune role of macrophages, encompassing alternative pyroptosis pathways, may also contribute to the worsening of liver IRI (107). This suggests that inhibiting the ultimate common step of pyroptosis, GSDMD (the key executor of cell membrane pore formation), may be a more suitable approach for targeted inhibition of pyroptosis (108). Caspases cleave GSDMD, releasing the N-terminal p30 fragment (GSDMD-NT), which oligomerizes and forms pores on the cell membrane to induce pyroptosis (43). Directly inhibiting the oligomerization of GSDMD-NT is undoubtedly the most direct way to inhibit pyroptosis, and many studies have explored this avenue. Disulfide bonds are crucial for the oligomerization of GSDMD-NT. Through screening, a small molecule inhibitor, necrosulfonamide (NSA), was identified. NSA disrupts the disulfide bonds formed by Cys through its interaction with MLKL. Research has revealed that NSA can directly bind to GSDMD, inhibiting the oligomerization of GSDMD-NT. Moreover, NSA is capable of blocking the release of IL-1β in monocyte/macrophage pyroptosis and extends the survival period of mice in a sepsis model (109). Fumaric acid, an intermediate product accumulated in the Krebs cycle during aerobic glycolysis in activated macrophages, also influences the oligomerization of GSDMD-NT. Cys192 (Cys191 in humans) is the site for the oligomerization of GSDMD-NT. Treatment of recombinant human or mouse GSDMD with Monomethyl fumarate (MMF) leads to abundant monomethyl succinate (2-methylsuccinyl cysteine) at Cys191 in human and Cys192 in mouse, respectively. The succinylation of GSDMD (an irreversible post-translational modification) limits its processing and oligomerization, thereby impeding its interaction with caspase and its ability to induce pyroptosis. Targeting the cysteine residues Cys191/Cys192 in GSDMD, the drug candidate dimethyl fumarate (DMF), a fumaric acid salt derivative, has received approval from the United States Food and Drug Administration (FDA) for the treatment of multiple sclerosis (MS). MS patients treated with DMF demonstrate notably decreased levels of IL-1β and GSDMD-NT in peripheral blood mononuclear cells (PBMCs) (110). In other inflammatory diseases, such as familial Mediterranean fever (FMF) caused by constitutive activation of the Pyrin inflammasome, DMF administration similarly alleviated pathological manifestations in FMF mice, including the secretion of IL-1β and the formation of GSDMD-NT (111). Similarly, high-throughput biochemical screening identified disulfiram as an effective inhibitor of GSDMD pore formation, and it has been demonstrated that disulfiram deactivates reactive Cys residues through covalent modification (112). In mice with LPS-induced sepsis, disulfiram inhibits the oligomerization of GSDMD-NT by covalently modifying Cys191, thereby suppressing macrophage pyroptosis and alleviating the severity of inflammation (113).The targeted therapeutic strategy inhibiting GSDMD-NT oligomerization has also demonstrated efficacy in liver diseases. Phenethyl isothiocyanate (PEITC) directly inhibits GSDMD-NT by interacting with Cys191, thereby suppressing pyroptosis. PEITC mitigates liver damage induced by ConA and CCl4 through the inhibition of hepatocyte pyroptosis. However, whether PEITC similarly inhibits GSDMD in liver macrophages has not been investigated (114). Inhibiting Caspase-1-GSDMD in innate immune cells of the liver, rather than in hepatocytes, significantly improves liver damage during liver IRI (57). The succinylation of GSDMD in macrophages appears to be an effective means of inhibiting cell pyroptosis in AIH. Research suggests that DMF promotes the phosphorylation of specific serine/threonine residues on NLRP3 by PKA, thereby inhibiting inflammasome activation, GSDMD-N formation, and cell pyroptosis. Although not explicitly analyzed in the research, it is believed that this inhibitory effect may be achieved to some extent through the suppression of GSDMD oligomerization (115). Another study indicates that NSA inhibits the pyroptosis pathway in ALF, leading to an increased survival rate in ALF mice. However, the study did not determine whether these pyroptotic cells are primarily hepatocytes or macrophages (116). Therefore, targeting GSDMD-NT oligomerization has shown promising results in inflammatory and autoimmune liver diseases. Given the crucial role of macrophages in the immune response and pyroptosis in such liver diseases, further exploration of specific targeting of macrophage GSDMD-NT deserves attention in subsequent studies. Furthermore, in Caspase-3-mediated pyroptosis, cells with high expression of GSDME undergo pyroptosis upon apoptotic stimulation induced by chemotherapy drugs (30). Competitive inhibition of caspase-3 using an inhibitor, a GSDME-derived peptide targeting the caspase-3 cleavage site, effectively suppresses chemotherapy-induced apoptosis and pyroptosis. Moreover, this derived peptide can inhibit apoptosis and pyroptosis in hepatocytes and macrophages, thereby alleviating liver damage induced by deoxycholic acid and BDL in mice (117). This research on GSDME-derived peptides provides an innovative direction for future therapies targeting macrophage pyroptosis. However, high concentrations of the derived peptide may also target other cysteine proteases. Therefore, addressing the challenge of improving the peptide’s specificity to mitigate off-target effects warrants further attention in subsequent research.

## 7 Conclusion

Pyroptosis, an inflammation-associated programmed cell death, serves as an “alarm” by propagating inflammation to the surrounding cellular milieu. This phenomenon was initially identified in macrophages infected with gram-negative bacteria. Remarkably, macrophages are integral components of the human innate immune system, especially within the liver, where they constitute approximately 90% of the total macrophage population in the body. They are involved in various bacterial and aseptic inflammatory liver injuries. This comprehensive review highlights the multifaceted role of macrophage pyroptosis in the development and progression of liver diseases such as ALI, IRI, NAFLD, ALD, HCC, liver failure, and fibrosis. Investigating methods to modulate macrophage pyroptosis as a strategic approach to devising innovative and effective treatments for liver diseases is a prominent avenue of ongoing research.

## Author contributions

LN: Writing – original draft. DC: Writing – original draft, Formal analysis. YZ: Conceptualization, Writing – review & editing. RY: Data curation, Writing – original draft. PF: Conceptualization, Methodology, Writing – review & editing.

## References

[1] Kubes P Jenne C. Immune responses in the liver. Annu Rev Immunol (2018) 36:247–77. doi: 10.1146/annurev-immunol-051116-052415 29328785

[2] Pol S Lagaye S. The remarkable history of the hepatitis C virus. Genes Immun (2019) 20(5):436–46. doi: 10.1038/s41435-019-0066-z 31019253

[3] Wong RJ Kaufman HW Niles JK Kapoor H Gish RG. Simplifying treatment criteria in chronic hepatitis B: reducing barriers to elimination. Clin Infect Dis (2023) 76(3):e791–800. doi: 10.1093/cid/ciac385 35594550

[4] Asrani SK Devarbhavi H Eaton J Kamath PS. Burden of liver diseases in the world. J Hepatol (2019) 70(1):151–71. doi: 10.1016/j.jhep.2018.09.014 30266282

[5] Tacke F. Targeting hepatic macrophages to treat liver diseases. J Hepatol (2017) 66(6):1300–12. doi: 10.1016/j.jhep.2017.02.026 28267621

[6] Blériot C Chakarov S Ginhoux F. Determinants of resident tissue macrophage identity and function. Immun (2020) 52(6):957–70. doi: 10.1016/j.immuni.2020.05.014 32553181

[7] Cheng ML Nakib D Perciani CT MacParland SA. The immune niche of the liver. Clin Sci (Lond) (2021) 135(20):2445–66. doi: 10.1042/CS20190654 34709406

[8] Wang C Ma C Gong L Guo Y Fu K Zhang Y. Macrophage polarization and its role in liver disease. Front Immunol (2021) 12:803037. doi: 10.3389/fimmu.2021.803037 34970275 PMC8712501

[9] Guillot A Tacke F. Liver macrophages: old dogmas and new insights. Hepatol Commun (2019) 3(6):730–43. doi: 10.1002/hep4.1356 PMC6545867 31168508

[10] Knolle PA Uhrig A Protzer U Trippler M Duchmann R Meyer zum Büschenfelde KH. Interleukin-10 expression is autoregulated at the transcriptional level in human and murine Kupffer cells. Hepatol (1998) 27(1):93–9. doi: 10.1002/hep.510270116 9425923

[11] Mass E Ballesteros I Farlik M Halbritter F Günther P Crozet L. Specification of tissue-resident macrophages during organogenesis. Sci (2016) 353(6304). doi: 10.1126/science.aaf4238 PMC5066309 27492475

[12] Li W Chang N Li L. Heterogeneity and function of kupffer cells in liver injury. Front Immunol (2022) 13:940867. doi: 10.3389/fimmu.2022.940867 35833135 PMC9271789

[13] Elchaninov A Vishnyakova P Menyailo E Sukhikh G Fatkhudinov T. An eye on kupffer cells: development, phenotype and the macrophage niche. Int J Mol Sci (2022) 23(17). doi: 10.3390/ijms23179868 PMC9456487 36077265

[14] Musrati MA De Baetselier P Movahedi K Van Ginderachter JA. Ontogeny, functions and reprogramming of Kupffer cells upon infectious disease. Front Immunol (2023) 14:1238452. doi: 10.3389/fimmu.2023.1238452 37691953 PMC10485603

[15] Guilliams M Thierry GR Bonnardel J Bajenoff M. Establishment and maintenance of the macrophage niche. Immun (2020) 52(3):434–51. doi: 10.1016/j.immuni.2020.02.015 32187515

[16] Li W Yang Y Yang L Chang N Li L. Monocyte-derived Kupffer cells dominate in the Kupffer cell pool during liver injury. Cell Rep (2023) 42(10):113164. doi: 10.1016/j.celrep.2023.113164 37740916

[17] Troutman TD Kofman E Glass CK. Exploiting dynamic enhancer landscapes to decode macrophage and microglia phenotypes in health and disease. Mol Cell (2021) 81(19):3888–903. doi: 10.1016/j.molcel.2021.08.004 PMC8500948 34464593

[18] Liu Y Xiao J Cai J Li R Sui X Zhang J. Single-cell immune profiling of mouse liver aging reveals Cxcl2+ macrophages recruit neutrophils to aggravate liver injury. Hepatol (2023). doi: 10.1097/HEP.0000000000000590 PMC10871588 37695548

[19] van der Heide D Weiskirchen R Bansal R. Therapeutic targeting of hepatic macrophages for the treatment of liver diseases. Front Immunol (2019) 10:2852. doi: 10.3389/fimmu.2019.02852 31849997 PMC6901832

[20] Wang J Kubes P. A reservoir of mature cavity macrophages that can rapidly invade visceral organs to affect tissue repair. Cell (2016) 165(3):668–78. doi: 10.1016/j.cell.2016.03.009 27062926

[21] Zhang S Wan D Zhu M Wang G Zhang X Huang N. CD11b + CD43 hi Ly6C lo splenocyte-derived macrophages exacerbate liver fibrosis via spleen-liver axis. Hepatol (2023) 77(5):1612–29. doi: 10.1002/hep.32782 PMC10113005 36098707

[22] Genshaft AS Subudhi S Keo A Sanchez Vasquez JD Conceição-Neto N Mahamed D. Single-cell RNA sequencing of liver fine-needle aspirates captures immune diversity in the blood and liver in chronic hepatitis B patients. Hepatol (2023) 78(5):1525–41. doi: 10.1097/HEP.0000000000000438 PMC10581444 37158243

[23] Bennett H Troutman TD Zhou E Spann NJ Link VM Seidman JS. Discrimination of cell-intrinsic and environment-dependent effects of natural genetic variation on Kupffer cell epigenomes and transcriptomes. Nat Immunol (2023) 24(11):1825–38. doi: 10.1038/s41590-023-01631-w PMC10602851 37735593

[24] Shi J Zhao Y Wang K Shi X Wang Y Huang H. Cleavage of GSDMD by inflammatory caspases determines pyroptotic cell death. Nature (2015) 526(7575):660–5. doi: 10.1038/nature15514 26375003

[25] Zychlinsky A Prevost MC Sansonetti PJ. Shigella flexneri induces apoptosis in infected macrophages. Nature (1992) 358(6382):167–9. doi: 10.1038/358167a0 1614548

[26] Monack DM Detweiler CS Falkow S. Salmonella pathogenicity island 2-dependent macrophage death is mediated in part by the host cysteine protease caspase-1. Cell Microbiol (2001) 3(12):825–37. doi: 10.1046/j.1462-5822.2001.00162.x 11736994

[27] Fink SL Cookson BT. Apoptosis, pyroptosis, and necrosis: mechanistic description of dead and dying eukaryotic cells. Infect Immun (2005) 73(4):1907–16. doi: 10.1128/IAI.73.4.1907-1916.2005 PMC1087413 15784530

[28] Yu P Zhang X Liu N Tang L Peng C Chen X. Pyroptosis: mechanisms and diseases. Signal Transduct Target Ther (2021) 6(1):128. doi: 10.1038/s41392-021-00507-5 33776057 PMC8005494

[29] Shi J Gao W Shao F. Pyroptosis: gasdermin-mediated programmed necrotic cell death. Trends Biochem Sci (2017) 42(4):245–54. doi: 10.1016/j.tibs.2016.10.004 27932073

[30] Wang Y Gao W Shi X Ding J Liu W He H. Chemotherapy drugs induce pyroptosis through caspase-3 cleavage of a gasdermin. Nature (2017) 547(7661):99–103. doi: 10.1038/nature22393 28459430

[31] Hu L Chen M Chen X Zhao C Fang Z Wang H. Chemotherapy-induced pyroptosis is mediated by BAK/BAX-caspase-3-GSDME pathway and inhibited by 2-bromopalmitate. Cell Death Dis (2020) 11(4):281. doi: 10.1038/s41419-020-2476-2 32332857 PMC7181755

[32] Zhai Z Yang F Xu W Han J Luo G Li Y. Attenuation of rheumatoid arthritis through the inhibition of tumor necrosis factor-induced caspase 3/gasdermin E-mediated pyroptosis. Arthritis Rheumatol (2022) 74(3):427–40. doi: 10.1002/art.41963 PMC9305212 34480835

[33] Wu J Lin S Chen W Lian G Wu W Chen A. TNF-α contributes to sarcopenia through caspase-8/caspase-3/GSDME-mediated pyroptosis. Cell Death Discov (2023) 9(1):76. doi: 10.1038/s41420-023-01365-6 36823174 PMC9950087

[34] Liu Y Fang Y Chen X Wang Z Liang X Zhang T. Gasdermin E-mediated target cell pyroptosis by CAR T cells triggers cytokine release syndrome. Sci Immunol (2020) 5(43). doi: 10.1126/sciimmunol.aax7969 31953257

[35] Zhang Z Zhang Y Xia S Kong Q Li S Liu X. Gasdermin E suppresses tumour growth by activating anti-tumour immunity. Nature (2020) 579(7799):415–20. doi: 10.1038/s41586-020-2071-9 PMC7123794 32188940

[36] Zhou Z He H Wang K Shi X Wang Y Su Y. Granzyme A from cytotoxic lymphocytes cleaves GSDMB to trigger pyroptosis in target cells. Sci (2020) 368(6494). doi: 10.1126/science.aaz7548 32299851

[37] Kong Q Xia S Pan X Ye K Li Z Li H. Alternative splicing of GSDMB modulates killer lymphocyte-triggered pyroptosis. Sci Immunol (2023) 8(82):eadg3196. doi: 10.1126/sciimmunol.adg3196 37115914 PMC10338320

[38] Zielinski CE Mele F Aschenbrenner D Jarrossay D Ronchi F Gattorno M. Pathogen-induced human TH17 cells produce IFN-γ or IL-10 and are regulated by IL-1β. Nature (2012) 484(7395):514–8. doi: 10.1038/nature10957 22466287

[39] Bachmann M Pfeilschifter J Mühl H. A prominent role of interleukin-18 in acetaminophen-induced liver injury advocates its blockage for therapy of hepatic necroinflammation. Front Immunol (2018) 9:161. doi: 10.3389/fimmu.2018.00161 29472923 PMC5809456

[40] Finotto S Siebler J Hausding M Schipp M Wirtz S Klein S. Severe hepatic injury in interleukin 18 (IL-18) transgenic mice: a key role for IL-18 in regulating hepatocyte apoptosis. vivo Gut (2004) 53(3):392–400. doi: 10.1136/gut.2003.018572 14960523 PMC1773961

[41] Luan J Zhang X Wang S Li Y Fan J Chen W. NOD-like receptor protein 3 inflammasome-dependent IL-1β Accelerated conA-induced hepatitis. Front Immunol (2018) 9:758. doi: 10.3389/fimmu.2018.00758 29692782 PMC5902503

[42] Antunes MM Araújo AM Diniz AB Pereira RVS Alvarenga DM David BA. IL-33 signalling in liver immune cells enhances drug-induced liver injury and inflammation. Inflamm Res (2018) 67(1):77–88. doi: 10.1007/s00011-017-1098-3 29032512

[43] Liu X Zhang Z Ruan J Pan Y Magupalli VG Wu H. Inflammasome-activated gasdermin D causes pyroptosis by forming membrane pores. Nature (2016) 535(7610):153–8. doi: 10.1038/nature18629 PMC5539988 27383986

[44] Zhang Q Raoof M Chen Y Sumi Y Sursal T Junger W. Circulating mitochondrial DAMPs cause inflammatory responses to injury. Nature (2010) 464(7285):104–7. doi: 10.1038/nature08780 PMC2843437 20203610

[45] Mihm S. Danger-associated molecular patterns (DAMPs): molecular triggers for sterile inflammation in the liver. Int J Mol Sci (2018) 19(10). doi: 10.3390/ijms19103104 PMC6213769 30309020

[46] Chen M Zhang C Zhang J Kai G Lu B Huang Z. The involvement of DAMPs-mediated inflammation in cyclophosphamide-induced liver injury and the protection of liquiritigenin and liquiritin. Eur J Pharmacol (2019) 856:172421. doi: 10.1016/j.ejphar.2019.172421 31136760

[47] Woolbright BL Jaeschke H. Role of the inflammasome in acetaminophen-induced liver injury and acute liver failure. J Hepatol (2017) 66(4):836–48. doi: 10.1016/j.jhep.2016.11.017 PMC5362341 27913221

[48] Huebener P Pradere J-P Hernandez C Gwak G-Y Caviglia JM Mu X. The HMGB1/RAGE axis triggers neutrophil-mediated injury amplification following necrosis. J Clin Invest (2019) 130(4):1802. doi: 10.1172/JCI126976 PMC6436859 30829651

[49] Hou W Wei X Liang J Fang P Ma C Zhang Q. HMGB1-induced hepatocyte pyroptosis expanding inflammatory responses contributes to the pathogenesis of acute-on-chronic liver failure (ACLF). J Inflamm Res (2021) 14:7295–313. doi: 10.2147/JIR.S336626 PMC8711847 34992418

[50] Denning N-L Aziz M Gurien SD Wang P. DAMPs and NETs in sepsis. Front Immunol (2019) 10:2536. doi: 10.3389/fimmu.2019.02536 31736963 PMC6831555

[51] Lu L Zhou H Ni M Wang X Busuttil R Kupiec-Weglinski J. Innate immune regulations and liver ischemia-reperfusion injury. Transplantation (2016) 100(12):2601–10. doi: 10.1097/TP.0000000000001411 PMC5141614 27861288

[52] de Carvalho Ribeiro M Szabo G. Role of the inflammasome in liver disease. Annu Rev Pathol (2022) 17:345–65. doi: 10.1146/annurev-pathmechdis-032521-102529 PMC10501045 34752711

[53] Huang Y Zang K Shang F Guo S Gao L Zhang X. HMGB1 mediates acute liver injury in sepsis through pyroptosis of liver macrophages. Int J Burns Trauma (2020) 10(3):60–7. PMC7364415 32714629

[54] Luo L Fang Y Yuan Q Liao J Zhang Z. LPS activated macrophages induced hepatocyte pyroptosis via P2X7R activation of NLRP3 in mice. Iran J Immunol (2022) 19(1):4. doi: 10.22034/IJI.2022.90579.2016 35293345

[55] Yang W Tao K Zhang P Chen X Sun X Li R. Maresin 1 protects against lipopolysaccharide/d-galactosamine-induced acute liver injury by inhibiting macrophage pyroptosis and inflammatory response. Biochem Pharmacol (2022) 195:114863. doi: 10.1016/j.bcp.2021.114863 34861244

[56] Han C Pei H Sheng Y Wang J Zhou X Li W. Toxicological mechanism of triptolide-induced liver injury: Caspase3-GSDME-mediated pyroptosis of Kupffer cell. Ecotoxicol Environ Saf (2023) 258:114963. doi: 10.1016/j.ecoenv.2023.114963 37130490

[57] Li J Zhao J Xu M Li M Wang B Qu X. Blocking GSDMD processing in innate immune cells but not in hepatocytes protects hepatic ischemia-reperfusion injury. Cell Death Dis (2020) 11(4):244. doi: 10.1038/s41419-020-2437-9 32303674 PMC7165177

[58] Lu J Wang X Feng Z Chen Y Wen D Liu Z. The protective effect of isoflurane pretreatment on liver IRI by suppressing noncanonical pyroptosis of liver macrophages. Int Immunopharmacol (2021) 99:107977. doi: 10.1016/j.intimp.2021.107977 34332342

[59] Sheng M Weng Y Cao Y Zhang C Lin Y Yu W. Caspase 6/NR4A1/SOX9 signaling axis regulates hepatic inflammation and pyroptosis in ischemia-stressed fatty liver. Cell Death Discov (2023) 9(1):106. doi: 10.1038/s41420-023-01396-z 36977670 PMC10043527

[60] Zou Z Shang R Zhou L Du D Yang Y Xie Y. The novel myD88 inhibitor TJ-M2010-5 protects against hepatic ischemia-reperfusion injury by suppressing pyroptosis in mice. Transplantation (2023) 107(2):392–404. doi: 10.1097/TP.0000000000004317 36226835 PMC9875839

[61] Hua S Ma M Fei X Zhang Y Gong F Fang M. Glycyrrhizin attenuates hepatic ischemia-reperfusion injury by suppressing HMGB1-dependent GSDMD-mediated kupffer cells pyroptosis. Int Immunopharmacol (2019) 68:145–55. doi: 10.1016/j.intimp.2019.01.002 30634142

[62] Wu J Liu Q Zhang X Wu X Zhao Y Ren J. STING-dependent induction of lipid peroxidation mediates intestinal ischemia-reperfusion injury. Free Radic Biol Med (2021) 163:135–40. doi: 10.1016/j.freeradbiomed.2020.12.010 33347986

[63] Kadono K Kageyama S Nakamura K Hirao H Ito T Kojima H. Myeloid Ikaros-SIRT1 signaling axis regulates hepatic inflammation and pyroptosis in ischemia-stressed mouse and human liver. J Hepatol (2022) 76(4):896–909. doi: 10.1016/j.jhep.2021.11.026 34871625 PMC9704689

[64] Jiang X Gu Y Huang Y Zhou Y Pang N Luo J. CBD alleviates liver injuries in alcoholics with high-fat high-cholesterol diet through regulating NLRP3 inflammasome-pyroptosis pathway. Front Pharmacol (2021) 12:724747. doi: 10.3389/fphar.2021.724747 34630100 PMC8493333

[65] Shu B Zhou Y-X Li H Zhang R-Z He C Yang X. The METTL3/MALAT1/PTBP1/USP8/TAK1 axis promotes pyroptosis and M1 polarization of macrophages and contributes to liver fibrosis. Cell Death Discov (2021) 7(1):368. doi: 10.1038/s41420-021-00756-x 34839365 PMC8627510

[66] Wree A McGeough MD Peña CA Schlattjan M Li H Inzaugarat ME. NLRP3 inflammasome activation is required for fibrosis development in NAFLD. J Mol Med (Berl) (2014) 92(10):1069–82. doi: 10.1007/s00109-014-1170-1 PMC4349416 24861026

[67] Dixon LJ Flask CA PapouChado BG Feldstein AE Nagy LE. Caspase-1 as a central regulator of high fat diet-induced non-alcoholic steatohepatitis. PloS One (2013) 8(2):e56100. doi: 10.1371/journal.pone.0056100 23409132 PMC3567081

[68] Drummer CT Saaoud F Jhala NC Cueto R Sun Y Xu K. Caspase-11 promotes high-fat diet-induced NAFLD by increasing glycolysis, OXPHOS, and pyroptosis in macrophages. Front Immunol (2023) 14:1113883. doi: 10.3389/fimmu.2023.1113883 36776889 PMC9909353

[69] Zhang K Shi Z Zhang M Dong X Zheng L Li G. Silencing lncRNA Lfar1 alleviates the classical activation and pyoptosis of macrophage in hepatic fibrosis. Cell Death Dis (2020) 11(2):132. doi: 10.1038/s41419-020-2323-5 32071306 PMC7028920

[70] Liu M He J Zheng S Zhang K Ouyang Y Zhang Y. Human umbilical cord mesenchymal stem cells ameliorate acute liver failure by inhibiting apoptosis, inflammation and pyroptosis. Ann Transl Med (2021) 9(21):1615. doi: 10.21037/atm-21-2885 34926659 PMC8640895

[71] Bai L Lu W Tang S Tang H Xu M Liang C. Galectin-3 critically mediates the hepatoprotection conferred by M2-like macrophages in ACLF by inhibiting pyroptosis but not necroptosis signalling. Cell Death Dis (2022) 13(9):775. doi: 10.1038/s41419-022-05181-1 36075893 PMC9458748

[72] Zheng Y Cui B Sun W Wang S Huang X Gao H. Potential crosstalk between liver and extra-liver organs in mouse models of acute liver injury. Int J Biol Sci (2020) 16(7):1166–79. doi: 10.7150/ijbs.41293 PMC7053327 32174792

[73] Guo H Sun J Li D Hu Y Yu X Hua H. Shikonin attenuates acetaminophen-induced acute liver injury via inhibition of oxidative stress and inflammation. BioMed Pharmacother (2019) 112:108704. doi: 10.1016/j.biopha.2019.108704 30818140

[74] Tsutsui H Nishiguchi S. Importance of Kupffer cells in the development of acute liver injuries in mice. Int J Mol Sci (2014) 15(5):7711–30. doi: 10.3390/ijms15057711 PMC4057701 24802875

[75] Strnad P Tacke F Koch A Trautwein C. Liver - guardian, modifier and target of sepsis. Nat Rev Gastroenterol Hepatol (2017) 14(1):55–66. doi: 10.1038/nrgastro.2016.168 27924081

[76] Zhai Y Petrowsky H Hong JC Busuttil RW Kupiec-Weglinski JW. Ischaemia-reperfusion injury in liver transplantation–from bench to bedside. Nat Rev Gastroenterol Hepatol (2013) 10(2):79–89. doi: 10.1038/nrgastro.2012.225 23229329 PMC3577927

[77] Dar WA Sullivan E Bynon JS Eltzschig H Ju C. Ischaemia reperfusion injury in liver transplantation: Cellular and molecular mechanisms. Liver Int (2019) 39(5):788–801. doi: 10.1111/liv.14091 30843314 PMC6483869

[78] Kolachala VL Lopez C Shen M Shayakhmetov D Gupta NA. Ischemia reperfusion injury induces pyroptosis and mediates injury in steatotic liver thorough Caspase 1 activation. Apoptosis (2021) 26(5-6):361–70. doi: 10.1007/s10495-021-01673-1 33990906

[79] Chen R-X Jiang W-J Liu S-C Wang Z-Y Wang Z-B Zhou T. Apolipoprotein A-1 protected hepatic ischaemia-reperfusion injury through suppressing macrophage pyroptosis via TLR4-NF-κB pathway. Liver Int (2023) 43(1):234–48. doi: 10.1111/liv.15448 36203339

[80] Wu X-Y Chen Y-J Liu C-A Gong J-H Xu X-S. STING induces liver ischemia-reperfusion injury by promoting calcium-dependent caspase 1-GSDMD processing in macrophages. Oxid Med Cell Longev (2022) 2022:8123157. doi: 10.1155/2022/8123157 35281468 PMC8906939

[81] Ambade A Lowe P Kodys K Catalano D Gyongyosi B Cho Y. Pharmacological inhibition of CCR2/5 signaling prevents and reverses alcohol-induced liver damage, steatosis, and inflammation in mice. Hepatol (2019) 69(3):1105–21. doi: 10.1002/hep.30249 PMC6393202 30179264

[82] Gao B Bataller R. Alcoholic liver disease: pathogenesis and new therapeutic targets. Gastroenterol (2011) 141(5):1572–85. doi: 10.1053/j.gastro.2011.09.002 PMC3214974 21920463

[83] Pan X-S Li B-W Wang L-L Li N Lin H-M Zhang J. Kupffer cell pyroptosis mediated by METTL3 contributes to the progression of alcoholic steatohepatitis. FASEB J (2023) 37(6):e22965. doi: 10.1096/fj.202300059RR 37171272

[84] Slevin E Baiocchi L Wu N Ekser B Sato K Lin E. Kupffer cells: inflammation pathways and cell-cell interactions in alcohol-associated liver disease. Am J Pathol (2020) 190(11):2185–93. doi: 10.1016/j.ajpath.2020.08.014 PMC7587925 32919978

[85] Heo MJ Kim TH You JS Blaya D Sancho-Bru P Kim SG. Alcohol dysregulates miR-148a in hepatocytes through FoxO1, facilitating pyroptosis via TXNIP overexpression. Gut (2019) 68(4):708–20. doi: 10.1136/gutjnl-2017-315123 PMC6581021 29475852

[86] Bank RA Zandstra J Room H Petersen AH van Putten SM. Biomaterial encapsulation is enhanced in the early stages of the foreign body reaction during conditional macrophage depletion in transgenic macrophage fas-induced apoptosis mice. Tissue Eng Part A (2017) 23(19-20):1078–87. doi: 10.1089/ten.TEA.2016.0499 28090808

[87] Riazi K Azhari H Charette JH Underwood FE King JA Afshar EE. The prevalence and incidence of NAFLD worldwide: a systematic review and meta-analysis. Lancet Gastroenterol Hepatol (2022) 7(9):851–61. doi: 10.1016/S2468-1253(22)00165-0 35798021

[88] Tilg H Adolph TE Dudek M Knolle P. Non-alcoholic fatty liver disease: the interplay between metabolism, microbes and immunity. Nat Metab (2021) 3(12):1596–607. doi: 10.1038/s42255-021-00501-9 34931080

[89] Krenkel O Tacke F. Liver macrophages in tissue homeostasis and disease. Nat Rev Immunol (2017) 17(5):306–21. doi: 10.1038/nri.2017.11 28317925

[90] Syn W-K Oo YH Pereira TA Karaca GF Jung Y Omenetti A. Accumulation of natural killer T cells in progressive nonalcoholic fatty liver disease. Hepatol (2010) 51(6):1998–2007. doi: 10.1002/hep.23599 PMC2920131 20512988

[91] Barreby E Chen P Aouadi M. Macrophage functional diversity in NAFLD - more than inflammation. Nat Rev Endocrinol (2022) 18(8):461–72. doi: 10.1038/s41574-022-00675-6 35534573

[92] Xu B Jiang M Chu Y Wang W Chen D Li X. Gasdermin D plays a key role as a pyroptosis executor of non-alcoholic steatohepatitis in humans and mice. J Hepatol (2018) 68(4):773–82. doi: 10.1016/j.jhep.2017.11.040 29273476

[93] Gaul S Leszczynska A Alegre F Kaufmann B Johnson CD Adams LA. Hepatocyte pyroptosis and release of inflammasome particles induce stellate cell activation and liver fibrosis. J Hepatol (2021) 74(1):156–67. doi: 10.1016/j.jhep.2020.07.041 PMC7749849 32763266

[94] Wree A Eguchi A McGeough MD Pena CA Johnson CD Canbay A. NLRP3 inflammasome activation results in hepatocyte pyroptosis, liver inflammation, and fibrosis in mice. Hepatol (2014) 59(3):898–910. doi: 10.1002/hep.26592 PMC4008151 23813842

[95] Zou H Li M Lei Q Luo Z Xue Y Yao D. Economic burden and quality of life of hepatocellular carcinoma in greater China: A systematic review. Front Public Health (2022) 10:801981. doi: 10.3389/fpubh.2022.801981 35530735 PMC9068962

[96] Raffetti E Portolani N Molfino S Mentasti S Baiocchi GL Magoni M. Is survival for hepatocellular carcinoma increasing? A population-based study on survival of hepatocellular carcinoma patients in the 1990s and 2000s. Clin Res Hepatol Gastroenterol (2021) 45(1):101433. doi: 10.1016/j.clinre.2020.04.004 32409284

[97] Ding T Xu J Wang F Shi M Zhang Y Li S-P. High tumor-infiltrating macrophage density predicts poor prognosis in patients with primary hepatocellular carcinoma after resection. Hum Pathol (2009) 40(3):381–9. doi: 10.1016/j.humpath.2008.08.011 18992916

[98] Schneider C Teufel A Yevsa T Staib F Hohmeyer A Walenda G. Adaptive immunity suppresses formation and progression of diethylnitrosamine-induced liver cancer. Gut (2012) 61(12):1733–43. doi: 10.1136/gutjnl-2011-301116 PMC4533880 22267597

[99] Capece D Fischietti M Verzella D Gaggiano A Cicciarelli G Tessitore A. The inflammatory microenvironment in hepatocellular carcinoma: a pivotal role for tumor-associated macrophages. BioMed Res Int (2013) 2013:187204. doi: 10.1155/2013/187204 23533994 PMC3591180

[100] Ju C Tacke F. Hepatic macrophages in homeostasis and liver diseases: from pathogenesis to novel therapeutic strategies. Cell Mol Immunol (2016) 13(3):316–27. doi: 10.1038/cmi.2015.104 PMC4856798 26908374

[101] Hu K Xu Z Yao L Yan Y Zhou L Li J. Integrated analysis of expression, prognostic value and immune infiltration of GSDMs in hepatocellular carcinoma. Aging (Albany NY) (2021) 13(21):24117–35. doi: 10.18632/aging.203669 PMC8610125 34731088

[102] Ferstl P Trebicka J. Acute decompensation and acute-on-chronic liver failure. Clin Liver Dis (2021) 25(2):419–30. doi: 10.1016/j.cld.2021.01.009 33838858

[103] Sarin SK Choudhury A. Acute-on-chronic liver failure: terminology, mechanisms and management. Nat Rev Gastroenterol Hepatol (2016) 13(3):131–49. doi: 10.1038/nrgastro.2015.219 26837712

[104] Triantafyllou E Woollard KJ McPhail MJW Antoniades CG Possamai LA. The role of monocytes and macrophages in acute and acute-on-chronic liver failure. Front Immunol (2018) 9:2948. doi: 10.3389/fimmu.2018.02948 30619308 PMC6302023

[105] Stack JH Beaumont K Larsen PD Straley KS Henkel GW Randle JCR. IL-converting enzyme/caspase-1 inhibitor VX-765 blocks the hypersensitive response to an inflammatory stimulus in monocytes from familial cold autoinflammatory syndrome patients. J Immunol (2005) 175(4):2630–4. doi: 10.4049/jimmunol.175.4.2630 16081838

[106] Jiao M Wang J Liu W Zhao X Qin Y Zhang C. VX-765 inhibits pyroptosis and reduces inflammation to prevent acute liver failure by upregulating PPARα expression. Ann Hepatol (2023) 28(3):101082. doi: 10.1016/j.aohep.2023.101082 36893888

[107] Dai Q Jiang W Liu H Qing X Wang G Huang F. Kupffer cell-targeting strategy for the protection of hepatic ischemia/reperfusion injury. Nanotechnol (2021) 32(26). doi: 10.1088/1361-6528/abde02 33472187

[108] He W-T Wan H Hu L Chen P Wang X Huang Z. Gasdermin D is an executor of pyroptosis and required for interleukin-1β secretion. Cell Res (2015) 25(12):1285–98. doi: 10.1038/cr.2015.139 PMC4670995 26611636

[109] Rathkey JK Zhao J Liu Z Chen Y Yang J Kondolf HC. Chemical disruption of the pyroptotic pore-forming protein gasdermin D inhibits inflammatory cell death and sepsis. Sci Immunol (2018) 3(26). doi: 10.1126/sciimmunol.aat2738 PMC6462819 30143556

[110] Humphries F Shmuel-Galia L Ketelut-Carneiro N Li S Wang B Nemmara VV. Succination inactivates gasdermin D and blocks pyroptosis. Sci (2020) 369(6511):1633–7. doi: 10.1126/science.abb9818 PMC8744141 32820063

[111] Kanneganti A Malireddi RKS Saavedra PHV Vande Walle L Van Gorp H Kambara H. GSDMD is critical for autoinflammatory pathology in a mouse model of Familial Mediterranean Fever. J Exp Med (2018) 215(6):1519–29. doi: 10.1084/jem.20172060 PMC5987922 29793924

[112] Castillo-Villanueva A Rufino-González Y Méndez S-T Torres-Arroyo A Ponce-Macotela M Martínez-Gordillo MN. Disulfiram as a novel inactivator of Giardia lamblia triosephosphate isomerase with antigiardial potential. Int J Parasitol Drugs Drug Resist (2017) 7(3):425–32. doi: 10.1016/j.ijpddr.2017.11.003 PMC5727346 29197728

[113] Hu JJ Liu X Xia S Zhang Z Zhang Y Zhao J. FDA-approved disulfiram inhibits pyroptosis by blocking gasdermin D pore formation. Nat Immunol (2020) 21(7):736–45. doi: 10.1038/s41590-020-0669-6 PMC7316630 32367036

[114] Wang J Shi K An N Li S Bai M Wu X. Direct inhibition of GSDMD by PEITC reduces hepatocyte pyroptosis and alleviates acute liver injury in mice. Front Immunol (2022) 13:825428. doi: 10.3389/fimmu.2022.825428 35173734 PMC8841757

[115] Shi F-L Ni S-T Luo S-Q Hu B Xu R Liu S-Y. Dimethyl fumarate ameliorates autoimmune hepatitis in mice by blocking NLRP3 inflammasome activation. Int Immunopharmacol (2022) 108:108867. doi: 10.1016/j.intimp.2022.108867 35605433

[116] Wu Y-L Ou W-J Zhong M Lin S Zhu Y-Y. Gasdermin D inhibitor necrosulfonamide alleviates lipopolysaccharide/D-galactosamine-induced acute liver failure in mice. J Clin Transl Hepatol (2022) 10(6):1148–54. doi: 10.14218/JCTH.2021.00560 PMC9634782 36381100

[117] Xu W-F Zhang Q Ding C-J Sun H-Y Che Y Huang H. Gasdermin E-derived caspase-3 inhibitors effectively protect mice from acute hepatic failure. Acta Pharmacol Sin (2021) 42(1):68–76. doi: 10.1038/s41401-020-0434-2 32457417 PMC7921426

